# Tibial tubercle avulsion fractures in school sports injury: A case report

**DOI:** 10.1016/j.ijscr.2019.03.017

**Published:** 2019-03-28

**Authors:** Abderrahim Zaizi, Tarik El Yaacoubi, Bouchaib Chafry, Mostapha Boussouga

**Affiliations:** Department of Orthopaedic Surgery & Traumatology II, Mohamed V Military Hospital, Faculty of Medicine and Pharmacy, Mohamed V University, Rabat 10000, Morocco

**Keywords:** Avulsion-fracture, Knee, Sport, Tibial tubercle

## Abstract

**Introduction:**

Most injuries in adolescent occur during school sports like volleyball, football or basketball. Tibial tubercle avulsion fractures (TTAF) are an unusual condition, resulting from a forced extension of the knee opposed to fixed leg.

**Presentation of the case:**

A 16 years old male was hurt during school basketball, X-rays displayed avulsion fracture of tibial tuberosity of left knee, the treatment was operative using two cancellous screws, results were good including complete knee mobility and early coming back to school sport at 6 months.

**Discussion:**

TTAF remains rare accounting for <3% of all epiphyseal injuries, it is frequent in teenage boys with open physis during school sport. We review the pathophysiology, mechanism, classification, diagnosis, and management of this injury.

**Conclusion:**

complications are occasional and functional recuperation is common after closed reduction and cast immobilization for slightly or no displaced fractures, otherwise open reduction and internal fixation for displaced fractures.

## Highlights

• Most injuries in school occur during sport. Avulsion fractures of the tibial tubercle are uncommon school sports injuries.
• X-ray is the key to diagnosis. Then CT scan is needed to evaluate the fracture extension to the articular joint.
• Many cases are misdiagnosed and progress to recurvatum deformity especially in open physis individuals after neglected tibial tuberosity fractures.
• These injuries cause significant disruption to school and sport, but fortunately, complications are rare and functional recovery is usually complete.

## 1 Background

Tibial tubercle avulsion fractures are exceptional, accounting for less than 1% of all physeal injuries. Watson-Jones classification modified by Ogden and colleagues’ is mostly used for such fractures.

We report a case occurring in an adolescent boy during school sport, reported in line with the SCARE criteria [1].

## 2 Case report

**Fig. 1:**
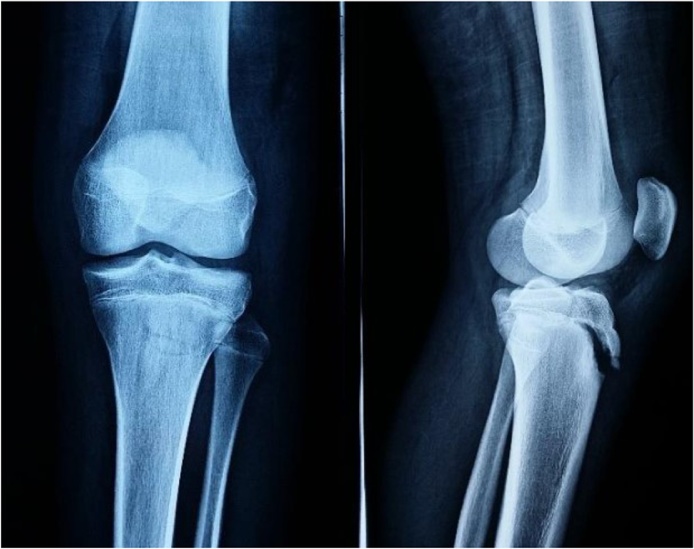
knee X-rays showing a displaced avulsion fracture of the tibial tuberosity.

**Fig. 2:**
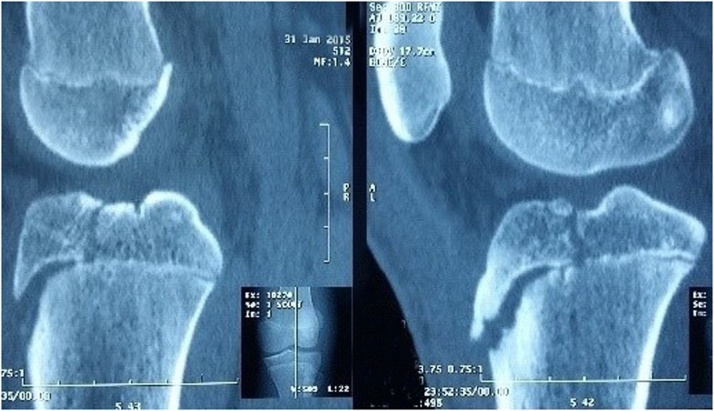
CT-scan showing a displaced avulsion fracture of the tibial tuberosity.

A 16 years old male was injured during school basketball when he touched the ground after jumping. He directly feels severe pain in his left knee and fell in terrain, he was admitted at the emergency department, the clinical examination of his left knee detected a flessum, swelling and exquisite pain of anterior tibial tuberosity with the inability to ambulate. X-rays showed a displaced avulsion fracture of tibial tuberosity (Fig. 1). A computerized tomography scan with 3D imaging demonstrated tibial tubercle avulsion fracture (Fig. 2) and categorized it Ogden Type III.

**Fig. 3:**
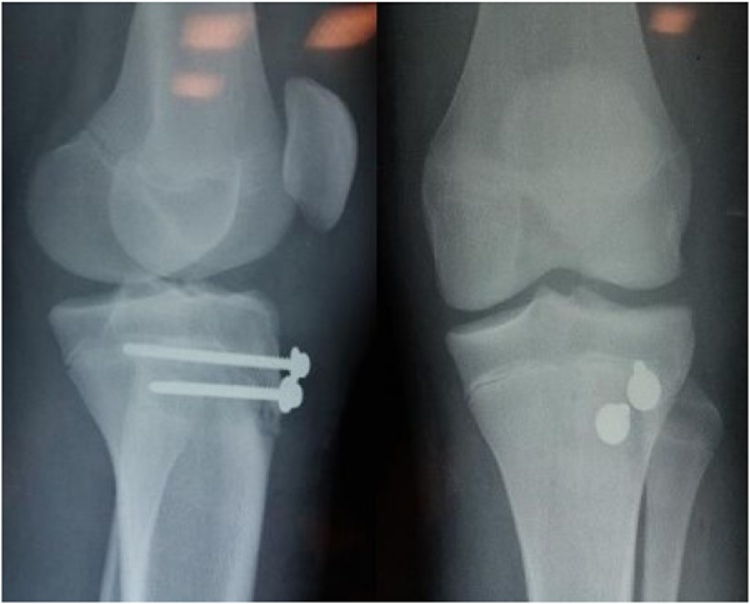
Post-operative X-rays showing a good reduction by two screws.

Operative intervention was achieved through open reduction and internal fixation via an anterior midline incision. By direct visualization, the tibial tubercle fragment was reduced manually and fixed using 2 cannulated screws with washers. Attention was taken to prevent splitting of tuberosity using small screws 3.5 mm. Post-operative X-rays showed a good reduction. (Fig. 3)

The operated limb was kept in full extension at cylinder cast for 4 weeks. At 4 additional weeks later, he began physiotherapy and prone active-knee flexion limited to 90°, with passive extension.

At 8 weeks, complete knee motion was authorized. At 3 months follow-up appointment, we notice radiographic union, and no clinical pain or limp, without any skeletal anomaly. Successful back to all normal activities including school sports, with a full range of knee mobility was attained at 6 months. At one-year follow-up, the alignment of lower limbs was preserved.

## 3 Discussion

School sport is not safety, 22% of teenagers could endure it [2]. Tibial tubercle avulsion fracture (TTAF) is a rare condition frequently occur in adolescent male, this stage of development corresponds to the pre-ossification period, trauma mechanism is a violent contraction of quadriceps during extension when jumping or instant knee ﬂexion opposed to quadriceps contraction during landing on the ground. [3] Predisposing factors comprise patella Baja, tight hamstrings, preexisting Osgood-Schlatter illness, and disorders including physeal anomalies [4].

Watson-Jones classification initially categorized TTAF into 3 types, Type I described an avulsion of the distal part of the tibial tubercle, distal to the proximal tibial physis. Type II prolonged across the physis but did not access the knee joint. Type III was an avulsion that continued proximal to the physis into the knee. This division was next modified by Ogden in 1980 by adding two groups A and B to designate comminution and displacement of the fragment. [5] Type-IV evoked by Ryu and Debenham describe avulsion fracture that extends posteriorly through the physis and may displace whole epiphysis and tubercle (Table 1). Frankl et al later purposed group-C concerning fractures associated with patella ligament avulsions [6]. and “Y” fracture defined type 5 by McKoy and Stanitski which matches to Type IIIB coupled to Type IV fracture forming a “Y” form. [7]

**Table 1:** Ogden classification of tibial tubercle avulsion fracture.

type	description
IA	Fracture distal to junction of ossifcation centre of proximal tibial epiphysis and tubercle
IB	Same as type IA but with comminution of fracture fragment
IIA	Fracture extension to junction of proximal tibial physis
IIB	Same as type IIA but with comminution of fracture fragment
IIIA	Fracture extends into joint through proximal tibial epiphysis with displacement of fracture fragment
IIIB	Same as type IIIA with comminution of fracture fragment Fracture extension transversely through proximal
IV	Tibial physis with displacement of fracture fragment

Type III lesions are mostly reported in the literature (as our case). Tibial tuberosity avulsion occurs frequently in association with Osgood disease, affecting anterior part of nucleus tuberosity. This disease happens subsequently to chronic pressure at tuberosal nucleus during teenage years producing minor tears and calcifications inside the nucleus itself. Otherwise, during TTAF unexpected quadriceps contraction affect deep part of proximal tibial growth cartilage. Therefore, chronic modification of nucleus tuberosity by Osgood disease can predispose to potential acute avulsion. [1,2,8]

Treatments of TTAF related in literature contrasts in line with avulsion fracture pattern: IA and IIA categories are operated within closed reduction and cast immobilization, a knee is kept in extension for 6 weeks, IIB and III AB categories are always managed surgically, then IB categories are often treated orthopedically, excepting cases of periosteum interpositions [9].

Anterior midline approaches are used to explore and reduce avulsed fragment, then fixation can be achieved by pins or screws, and strengthened by reparation of torn periosteum. Treatment devices continue innovation for skeletal injuries, such as arthroscopic assisted reduction of articular fractures and aiding osteosynthesis by means of several combinations; wires, screw, suture repairs, and tension band techniques permitting very good results [10].

## 4 Conclusion

TTAF is occasional, resulting in acute quadriceps contraction, it predominates in teenage boys with open physis during school sport. Treatment can be orthopedic or chirurgical according to lesion type. They are a source of school and sports interruption; however, complications are fortunately rare and functional recovery is usually complete.

## Conflict of interest

Authors report no conflicts of interest.

## Sources of funding

All authors disclose that this manuscript didn’t received no specific grant from any funding agency.

## Ethical approval

The study is exempt from ethnical approval in our institution.

This is a case report and the patient give us informed consent for publication.

## Consent

Parent gives informed consent for publication.

## Author contribution

Abderrahim Zaizi and Tarik El Yaacoubi make substantial contributions to acquisition of data, conception and design, and analysis and interpretation of data.

Bouchaib Chafry and Mostapha Boussouga participate in revising it critically for important intellectual content and give final approval of the version to be submitted.

## Registration of research studies

This case report don’t need to be registered because is not first-in-man.

## Guarantor

Abderrahim Zaizi and Tarik El Yaacoubi are the guarantor of this publication.

## Provenance and peer review

Not commissioned, externally peer reviewed.
